# Artificial Intelligence for Personalized Management of Acute Myeloid Leukemia

**DOI:** 10.3390/jpm16080397

**Published:** 2026-07-24

**Authors:** Pasquale Niscola, Valentina Gianfelici, Roberta Laureana, Marco Giovannini, Carla Mazzone, Fabio Efficace, Maria Ilaria Del Principe

**Affiliations:** 1 Hematology Unit, Medical Area Department, S. Eugenio Hospital (ASL Roma 2), Piazzale dell’Umanesimo 10, 00144 Rome, Italy; valentina.gianfelici@aslroma2.it (V.G.); roberta.laureana@aslroma2.it (R.L.); giovanninimd@libero.it (M.G.); carla.mazzone@aslroma2.it (C.M.); dlpmlr00@uniroma2.it (M.I.D.P.); 2 Italian Group for Adult Hematologic Diseases (GIMEMA), Data Center and Health Outcomes Research Unit, 00168 Rome, Italy; f.efficace@gimema.it; 3 Hematology Unit, Department of Biomedicine and Prevention, University Tor Vergata of Rome, 00133 Rome, Italy

**Keywords:** acute myeloid leukemia, artificial intelligence, convolutional neural networks, deep learning algorithms, diagnosis, fitness, genomic alterations, leukemia classification, machine learning, molecular testing, measurable residual disease, patient-reported outcomes, personalized medicine, prognostic value, quality of life, targeted therapies

## Abstract

Acute Myeloid Leukemia (AML) is a heterogeneous group of aggressive blood-related cancers that arise from the hematopoietic system, requiring specific treatments due to their genetic diversity and complexity. Artificial intelligence (AI) has emerged as a transformative technology in healthcare, with considerable potential to help manage AML. The application of AI approaches, such as Machine Learning (ML) models and Deep Learning (DL) algorithms, has been shown to aid risk stratification, diagnosis, treatment planning, and surveillance. This review highlights recent developments in AI applications for the personalized management of AML. We focus specifically on three major axes of personalization in AML: (1) the use of predictive models combining different data sources to improve prognostic assessment and guide risk-adaptive treatments; (2) prediction of treatment responses to various therapies based on data analysis; and (3) the use of AI for monitoring and adaptive trials in AML patients.

## 1. Introduction and Scope

Acute myeloid leukemia (AML) is a biologically diverse and complex group of hematopoietic malignancies characterized by the rapid proliferation of immature myeloid cells that do not differentiate properly [1,2]. Traditionally, treatment mainly involves uniform intensive chemotherapy (IC), notably the “7 + 3” induction protocol using cytarabine and anthracycline [1]. However, the modest long-term survival rate, about 25% to 30% in adults and under 15% in patients over 60, underscores the limitations of a one-size-fits-all approach [3]. AML shows substantial interpatient variability in genetics, epigenetics, microenvironmental interactions, and therapeutic response, ideally requiring a personalized and integrated treatment approach, considering the clinical and genomic disease features along with the patient’s fitness and values on expectations, values, and quality of life (QoL) issues [4,5,6]. Advances in high-throughput sequencing and molecular profiling have uncovered a wide range of genetic and epigenetic alterations, leading to a more personalized approach to treatment.

Within this rapidly evolving landscape, artificial intelligence (AI)-based approaches, including machine learning (ML) and deep learning (DL), are bringing novel advancements in the personalization of treatment for AML by unlocking the disease’s vast heterogeneity to help make better decisions about patients, predict risks, and tailor treatments to help those affected [7]. Present-day successful AML management is contingent upon an approach that spans from morphology-based examination at diagnosis through therapy-resistance prediction to checking minimal residual disease (MRD) [4]. Meanwhile, AI and ML/DL models enable doctors to move beyond mere reactive treatment toward proactive, highly tailored precision medicine [7,8,9,10,11,12]. With the help of AI, it will be possible to integrate multi-omics, imaging, cytometry, and clinical data at scales that are difficult to process manually. Thus, it will be possible to achieve more accurate risk stratification, predict drug response, and develop personalized treatment strategies. The literature indicates that AI may be used for diagnosis, prognosis, and treatment improvement for hematological malignancies and solid tumors, with specific applications in AML treatment [13,14,15,16,17,18,19]. While the role of AI in AML management holds remarkable promise, several challenges still stand in the way. They include heterogeneity in available data, the need for large, well-annotated datasets, the interpretability of AI models, and the integration of innovations into daily AML routines [7,8,9,10,11,12]. At the same time, the area is ripe with opportunities for federated learning to enable secure and efficient AML data exchange across various facilities and to support real-time bedside AI-assisted decision-making systems [7,8,9,10,11,12]. Collaborative effort from clinicians, data scientists, and governmental authorities is crucial to overcoming obstacles along the way. Thus, this review, aimed at a non-AI-expert audience, summarizes the role of AI in AML management and provides insight into how AI advances AML-related applications. As far as possible, AML-focused studies are analyzed to highlight methodological best practices and challenges related to AI application within the scope of interest, including the importance of data quality, interpretability, and generalizability, as well as regulatory considerations [13,15,18,19,20,21]. Undoubtedly, the advent of AI has brought tremendous improvements in diagnostic speed and predictivity [4]. However, implementing these innovations at the physician’s bedside requires further action from the scientific community. Currently, work is underway to overcome the “black box” obstacle by using Explainable AI. In addition, structural challenges in electronic health record interoperability and the lack of standardized datasets must be addressed to make these innovations a reality [7,8,9,10,11,12].

## 2. Methods

A thorough search of PubMed was conducted from database inception through May 2026. Both MeSH and non-MeSH terms related to AML, machine learning, deep learning, artificial intelligence, and diagnostics, including prognosis, survival rates, and risk stratification, were used. To set the context, relevant keywords and terms associated with AML were used in the PubMed search. The search process was flexible and iterative, focusing on English-language papers about AML and advanced personalized treatments.

## 3. Summary of Principles of the Different AI Approaches Applicable to the Management of AML

ML (Feature-Engineered) models include Decision-Tree Classifiers, Random Forests, and Support Vector Machines (SVMs). Decision-Tree Classifiers split data using a sequence of consecutive and nested “yes/no” questions that depend on data features. Just like in a flowchart, the data is split at each node until a final classification (the leaves) is reached. Moreover, like a single decision tree, it can be brittle and prone to overfitting. Random Forest constructs many decision trees, sometimes hundreds, using different data subsets, and then uses their votes to determine the correct output. Also, SVMs work by mapping data points into a high-dimensional space and finding the best boundary (a hyperplane) between the classes that maximizes the margin. DL (Representation Learning) includes Convolutional Neural Networks (CNNs) and Attention-Based Multi-Instance Learning (MIL) Models. CNNs are specifically designed to work with grid-like data, such as images. Instead of inspecting each pixel separately, the CNN passes small filters over the image (hence the term “convolution”) to detect local patterns, starting with simple edges and progressing to more complex visual structures. In addition, attention-based MIL Models are used when data is organized into “bags” (for example, a gigapixel medical scan consists of thousands of small tissue tiles). Nevertheless, you have information only about the whole bag, not its parts. In this way, the MIL component handles classifying the entire bag using all its instances. In contrast, the attention component automatically computes a weight score for each instance, thereby focusing on the most important ones [7,22].

### Algorithmic Approaches to ML and DL in AML

AI, including its subsets of ML and DL, offers a way to recognize complex patterns in large datasets and use them effectively, even when that complexity makes human analysis difficult. The creation of these models consists of several steps, starting with study design and proper implementation, including data collection, followed by data cleaning and preprocessing to convert the biological data into a computer-readable form [1]. Data pre-processing plays an integral role, as it involves selecting only the most important data for further processing and feature extraction, with consideration of the specific clinical question [1]. ML approaches are distinguished by learning paradigms, and the most common for the hematological classification task is supervised learning [22]. Training the model involves using labeled data to distinguish malignant from benign cell populations using algorithms such as SVMs, decision-tree classifiers, and random forests [22]. In contrast, since reinforcement learning uses feedback and a trial-and-error approach, it is well-suited to sequential problems and their outcomes [22]. However, DL techniques rely on hierarchical network architectures, such as convolutional neural networks, which have successfully found features in images without human supervision [22,23,24]. In general, AI in AML refers to the use of ML and DL in diagnostic, genetic, and prognostic processes. Table 1 presents taxonomic concepts of AI applicable to the personalized management of AML [7,8,22,25,26]. For these purposes, the disease needs to be properly diagnosed, with all its peculiarities taken into account [27,28,29]. Specifically, nowadays, the assessment of a patient’s fitness level in relation to therapy options is not limited to the traditional dichotomy “fit vs. unfit”. Instead, a patient is assessed for suitability for a particular treatment based on criteria such as age, performance status, and other factors [30]. Therefore, treatment choices depend on patient characteristics, disease-specific features, and the clinical response as assessed by MRD testing [31]. Furthermore, patient involvement in choosing treatment in accordance with their personal preferences and values, early symptom management, and essential QoL goals is increasingly important [4,8,30,32]. In this complex and continuing evolving scenario, prognostication has an essential role [33,34,35,36]. Indeed, treatment is driven by deep genomic sequencing to identify specific mutations. An ever-increasing number of tailor-made drugs with high clinical impact for specific patient categories carrying specific mutations are being developed to enrich the therapeutic arsenal for AML. Therefore, the treatment landscape of AML is constantly evolving, radically changing the traditional “one-size-fits-all” approach [4,8,37]. In this regard, AI and novel technologies will play an ever more extended role in prognostication and, in general, across all aspects of AML management, supporting the integration of precision medicine into standard practice in this setting [7,38]. Therefore, the treatment course in AML involves continuous evaluation of individual clinical features and personal performance abilities, thereby guiding incremental personalization, moving closer to the goal of controlling the disease, which has distinct fitness, social, biological, and treatment-specific signatures in each patient [4,8,30].

## 4. Paradigm Shift in AML Diagnostics & Classification

The shift in the paradigm of AML diagnosis and classification can be seen in the change from a morphology-centered, solely blast percent-dependent classification scheme to a genetics-based classification scheme. The current paradigm of diagnosing and classifying AML is based on WHO 5th Edition updates and the International Consensus Classification (ICC), along with risk stratification updates (European LeukemiaNet (ELN)). Before that, AML had been diagnosed based on 20% bone marrow or peripheral blood blasts. However, the modern paradigm focuses on the underlying biology rather than on a specific percentage used to diagnose AML. For cases of AML with recurrent genetic abnormalities (PML::RARA, RUNX1::RUNX1T1, CBFB::MYH11, KMT2A rearrangements), WHO abandoned the 20% blast criterion in the diagnosis of AML. ICC differs slightly from WHO in that it requires only a 10% blast percentage to classify as genetically defined AML. AML cases with 10–19% blasts lie at the biological boundary between MDS and AML. Changes in classification are evident in the increasing use of Next-Generation Sequencing (NGS) to group patients by their genotypic ontogeny (Myelodysplasia-Related AML (AML-MR), TP53 Mutated Entity). Now, AML-MR is not based on a subjective assessment of dysplasia using light microscopy but, rather, on cytogenetic abnormalities and new somatic mutations (ASXL1, BCOR, EZH2, SF3B1, SRSF2, STAG2, U2AF1, and ZRSR2). A particularly aggressive biology and poor prognosis to chemotherapeutic treatment are features of the TP53 Mutated Entity that ICC has established as a unique major category for AML with a TP53 mutation (20% blasts and variant allele fraction >10%). With the development of targeted agents, the “favorable”, “intermediate” and “adverse” risk definitions have become more flexible. In this regard, the current ELN guidelines classify the FLT3-ITD mutation as an intermediate, rather than an adverse, risk factor. This change is driven by the clinical success of integrating tyrosine kinase inhibitors (midostaurin, quizartinib) into upfront therapy. The favorable-risk group definition is now based on in-frame mutations in the bZIP region of the CEBPA gene, regardless of whether the mutations are monoallelic or biallelic. Thus, the new landscape dramatically changes the speed at which therapy is initiated. Treatment of AML is no longer based on the diagnosis of emergency due to an elevated blast count; rather, rapid NGS and cytogenetic testing are used to develop a targeted combination therapy (venetoclax regimen, menin inhibitors) from day one. Therefore, personalized management of AML involves therapy based on patients’ and disease characteristics, as well as genetic alterations [8,25,26]. For these purposes, the disease needs to be properly diagnosed, with all its peculiarities taken into account [27,28,29]. Specifically, nowadays, the assessment of a patient’s fitness level in relation to therapy options is not limited to the traditional dichotomy of “fit vs. unfit”. Instead, a patient is assessed for suitability for a particular treatment based on criteria such as age, performance status, and other factors [30]. Therefore, treatment choices depend on patient characteristics, disease-specific features, and the clinical response as assessed by MRD testing [31]. Furthermore, patient involvement in choosing treatment in accordance with their personal preferences and values, early symptom management, and essential QoL goals are increasingly important [4,8,30,32]. In this complex and continually evolving scenario, prognostication has an essential role [33,34,35,36]. Indeed, treatment is driven by deep genomic sequencing to identify specific mutations. An ever-increasing number of tailor-made drugs with high clinical impact for specific patient categories carrying specific mutations are being developed to enrich the therapeutic arsenal for AML. Therefore, the treatment landscape of AML is constantly evolving, radically changing the traditional “one-size-fits-all” approach [4,8,37]. In this regard, AI and novel technologies will play an ever more extended role in prognostication and, in general, across all aspects of AML management, supporting the integration of precision medicine into standard practice in this setting [7,38]. Therefore, the treatment course in AML involves continuous evaluation of individual clinical features and personal performance abilities, thereby guiding incremental personalization, moving closer to the goal of controlling the disease, which has distinct fitness, social, biological, and treatment-specific signatures in each patient [4,8,30].

### 4.1. AI-Assisted AML Diagnosis and Classification

For the diagnosis of AML, one should evaluate the disease’s morphological features and perform tests such as Multiparameter Flow Cytometry (MFC), cytogenetics, and genetic mutation testing. AI technology based on digital pathology offers the opportunity to diagnose AML with much higher accuracy than conventional methods. There is no doubt that the introduction of CNS algorithms has advanced digital pathology, as their use enables the classification of malignancies among white blood cells without the subjectivity and time required by microscopic analysis [23]. AI in diagnostic systems is equally effective at detecting various types of leukemia. The standard diagnostic techniques include visual examination of peripheral blood (PB) films and bone marrow (BM) smears by an expert hematopathologist, but they are subject to significant inter-observer variability. Consequently, AI-based technology could be much faster and more reliable for analyzing these samples [23,39]. Timely and accurate diagnosis of AML plays a vital role. It has been shown that the application of AI, namely ML and DL, is effective at processing vast datasets, including genomic, PB smears, and flow cytometry data, thereby transforming AML diagnosis and classification. The power of AI and ML enables the processing of high-dimensional data, such as genomics, transcriptomics, and epigenomics, achieving very high classification rates that surpass those of conventional microscopy and flow cytometric assays [39,40,41]. Also, ML and AI models that integrate multiple data sources, such as genomics, transcriptomics, and epigenomics, achieve high classification rates in molecular diagnostics [42].

### 4.2. AI-Based Advances in Precise Diagnosis in AML: Morphological Analysis and Digital Pathology

At first, in diagnosing AML, the difference between abnormal and healthy cells is determined morphologically using characteristics such as the large size of myeloid cells, an increased nuclear-to-cytoplasmic ratio, the presence of nucleoli, and the presence of Auer rods. DL methods, such as convolutional neural networks with layers of red-striped neurons modeled after human vision, enable the creation of solutions [43,44,45]. For this reason, AI models are highly effective at analyzing medical images and can detect, segment, and classify myeloid cells. Hence, current technology enables AI-driven models to conduct cytomorphological analysis at an expert level. For example, the DeepHeme framework matches hematopathologists’ analysis of BM aspirate samples with 95% accuracy, and AI models recognize and classify over 49 abnormal cell types in PB smear images with 89–92% precision [43,44,45]. Automation has made these processes even more efficient. For example, a recent approval of the Scopio Labs Full-Field BM™ app marks a significant moment in the history of AI-assisted BM smear analysis in practice [46]. Using DL technology, these algorithms identify regions of interest for differential cell counts and address the problem caused by the non-uniform distribution of cells in bone BM. Focusing on trails of cells following BM particles, where cells tend to be evenly distributed, models achieve higher-quality information necessary for accurate diagnostic classification [46]. In addition to diagnosing AML by PB and AML smears, AI can also predict genotypes by analyzing morphological phenotypes (Table 2) [47,48,49].

### 4.3. AI-Assisted Multiparameter Flow Cytometry

MFC techniques are pivotal for determining cell lineage and diagnosing aberrant immunophenotypes in AML [47]. Although MFC analysis is important, it still relies on expert analysis and manual gating, both of which are time-consuming and subjective, limiting the feasibility of these tasks [47,50,51]. New AI-based technologies help automate the complex process of cell classification. Whereas traditional analysis takes 10 to 20 min and requires multiple two-dimensional dot plots, the AI-assisted workflow reduces the time to approximately 1 min per case [52]. DL and ML can now identify normal hematopoietic subsets and distinguish leukemic blasts from non-leukemic mononuclear cells [46]. The introduction of advanced AI-based computational solutions to the MFC pipeline provides a multidimensional immunological assessment that is both reproducible and rapidly executable [50]. In this context, attention-based multi-instance learning models (ABMILMs) are among the most significant innovations in this area [50]. Such a DL architecture enables analysis of individual cellular events within the MFC dataset and the identification of relevant markers and event clusters for a given patient [50]. In addition, MFC can diagnose leukemic cells by searching for the “leukemia-associated immunophenotype”, which monitors the presence of marker combinations established during diagnosis, and the “difference from normal”, which detects deviations from well-described maturation pathways in healthy BM [51]. This technology is not only efficient for initial diagnosis but also for showing clinically significant molecular variants, such as t(15;17) or mutations in nucleophosmin1 (NPM1), before full sequencing results are available [46]. With prompt information on specific genomic alterations, AI-assisted flow cytometry enables targeted treatment to be administered much faster, within hours of sample collection [46]. On the other hand, this technique provides comprehensible information via attention values, revealing the most informative markers and thereby aiding hematopathologists in obtaining visual evidence of their work [50]. Another breakthrough is the ML-Guided AML MRD Analysis pipeline, which identifies populations of MRD and immature monocytic subsets that are very difficult to detect manually [53]. Importantly, MFC is critical for showing Leukemic Stem Cells (LSCs), whose presence is a leading cause of relapse in AML [4,54]. They are usually resistant to conventional IC and live in the BM microenvironment. AI-assisted MFC enables the detection and differentiation of rare, therapy-resistant LSCs that differ from normal Hematopoietic Stem Cells (HSCs) during AML diagnosis and monitoring [4,54]. Using markers such as CLEC12A, this technology distinguishes LSCs (CD34+, CD38−, CD45low, CD14−, CLEC12A+) from their healthy HSC counterparts with 93.42% precision, preventing false-negative results [55]. Table 3 presents some issues related to the performance of AI-assisted MFC.

### 4.4. AI-Assisted Genomics Detection in AML

The prognosis of AML is very uncertain, needing precise predictions in terms of treatment choice. Currently, molecular classification of AML is predominantly performed using NGS panels, whole-exome sequencing, and whole-genome sequencing. In this context, AI, ML, and DL have proven to be game changers. ML and DL excel at handling complex, high-dimensional multi-omics data, enabling the detection of hidden genetic and molecular alterations that standard methods might miss [37]. By serving as a bridge between NGS and clinical practice, AI-assisted tools are completely reshaping the entire spectrum of AML from molecular subtype and mutation prediction to survival prognosis and ultra-sensitive MRD. Genomic Detection of AML Using AI uses ML and DL techniques to interpret genomic data, thereby predicting gene mutations and assessing MRD [56,57]. By combining different types of omics data (DNA Methylation, RNA-seq, and genomics), subgroups of AML could be found [58]. This approach is capable not only of correct grouping but also of finding AML subgroups that are responsive to specific targeted inhibitors [58]. By analyzing large-scale ‘omics’ data from thousands of AML patients, AI has overcome human limitations in detecting complex, nonlinear interactions, discovering hidden patient subgroups, and showing metabolic and genetic targets of vulnerable cells. Instead of treating clinical data separately, AI uses advanced architectures to integrate distinct biological layers into a single computational representation. In this context, ML models detect complex co-occurrences of gene expression in AML, including, for example, the co-occurrence of NPM1 mutations with certain structural variants of FLT3-ITD and DNMT3A. Also, DL models detect mRNA and lncRNA expression levels in thousands simultaneously, using DNA methylation (epigenomic) patterns to classify rapid-onset variants of acute leukemia. Again, AI-assisted discovery of protein expression networks (e.g., anti-apoptotic blocks), metabolic outputs of leukemia cells, and nutrient dependencies of these cells enables improved risk stratification of AML. Finally, AI-assisted models detect critical biological pressure points. For example, overcoming venetoclax resistance and ex vivo mapping of drug sensitivity, along with genome sequencing, are among the remarkable applications of AI-assisted methods. In this regard, even though venetoclax-based treatments act through the BCL-2 pathway, resistance is widespread. AI-assisted proteogenomic analysis revealed that subgroups overexpress MCL-1 and depend on oxidative phosphorylation for survival; thus, combination therapy is needed. Again, different FLT3, CBFβ-MYH11, and NRAS sub-mutations correspond directly to targeted responses to kinase inhibitors, such as quizartinib [58]. The potential of NGS to detect MRD can also be enhanced by applying AI-driven genome analysis that saves significant time when working with complex NGS panels. In addition, such an approach helps make fast frontline therapeutic decisions, reduce intra-observer variation, and find rare resistant clones [56,57]. Alongside genome analysis, DL can help with automated karyotyping, improve the quality of metaphase scans, and aid in detecting cryptic chromosome aberrations [13]. In addition, ML algorithms trained on whole-genome sequencing and cytogenetic arrays can distinguish AML from MDS, as a small number of genetic markers have been shown to suffice to supersede traditional criteria based on blast count [59]. Nevertheless, there are some issues with the use of AI in AML genomics. First, AI needs large databases with rare variants and is prone to data bias; second, standardization of the regulatory framework for its use in practice is needed [13,37].

### 4.5. Classification and Morphology-to-Genotype (Morphogenetic Screening)

The classification of AML into different types is important for the right therapy. Thanks to ML and transcriptomics, this area has changed drastically. In this regard, DL methods, such as an attention-based multilayer perceptron, can be used to classify AML subtypes based on transcriptomics [60]. Notably, one clinical innovation in this area is the ability to perform image-to-genotype analysis, i.e., predicting somatic mutations from PB films and BM smears, bypassing NGS and its latency [47,48,49,50]. Predicting morphogenetic status is important because there are highly effective drugs to address the mutations [61,62,63,64,65]. This technique allows for morphogenetic screening before continuing to NGS. In addition, DL can predict multiple clinically relevant factors, such as fusion genes, cytogenetic risk, and mutation status, from single-cell smear images. In addition to classifying AML, AI can predict genotypes based on morphological phenotypes (Table 2). A prominent example was predicting the presence of *NPM1* mutations, the most common type of mutation in adult AML [9,62,66]. With the help of occlusion sensitivity maps, new characteristics of *NPM1* mutations, such as a perinuclear zone of lightening and specific patterns of condensed chromatin, were found, enabling predictions that would be more difficult for humans to make [9,62]. Moreover, morphological phenotype, by linking phenotype and genomics, enables prediction, saving time otherwise spent examining subtype samples, such as acute promyelocytic leukemia, when prompt medical intervention prevents deadly coagulopathy [42]. Therefore, AI-assisted morphological genotyping analysis has been reported to find genomic abnormalities, such as isocitrate dehydrogenase (*IDH1/2*), FMS-like tyrosine kinase 3-internal tandem duplication (*FLT3-ITD*), *NPM1*, Promyelocytic Leukemia and Retinoic Acid Receptor Alpha (*PML::RARA*), or runt-related transcription factor 1/*RUNX1* Partner Transcriptional Co-Repressor (*RUNX1::RUNX1T1*), by analyzing digital images of BM smears (Table 2). This method shows high predictive accuracy and provides a faster, more cost-effective alternative to traditional genetic testing for AML patients [47,48,49,61,62,63,64,65,66].

### 4.6. Potential Limitations and Concerns Related to AI Failure in Morphological Analysis or MFC Analysis

The methods used by artificial intelligence (AI) to analyze blood cells and MFC are highly effective, although they have some limitations. Indeed, there is no guarantee of the absolute accuracy of AI algorithms for morphological assessment and MFC reading. This issue may have several regulatory and compliance implications beyond the search for technical reasons for the error. The consequences of AI failure are rather diverse and include clinical, financial, psychological, and legal elements. AI failures directly affect the treatment path, potentially bringing unwanted consequences for patients. As far as clinical implications go, there are two types of AI errors: false negatives (failure to detect disease) and false positives (falsely alarming). The first is that the AI fails to recognize cancerous cells, abnormal cell shapes, or even abnormal flow cytometry readings, causing delays in the diagnosis of diseases such as AML. The latter is that AI diagnoses may produce false positives because the AI may mistake normal cell variations or technical glitches for cancerous cells, leading to overreliance on chemotherapy or bone marrow biopsies. Notably, in the leukemia setting, including AML, AI-assisted MFC helps detect very small disease samples, such as MRD, after treatment; if AI does not find them, doctors may not realize the cancer is still present, leading them to consider a patient in remission. Obviously, correcting misdiagnosis, retesting, and managing the adverse effects of unnecessary treatment contribute to increased medical expenses. Also, if clinicians lose confidence in AI due to a high number of errors, they can revert to manual counting, thereby losing the time-saving advantage of automation. It is important to mention that the potential diagnosis of AML because of the false positive results of AI is a great stressor and may cause the burnout of hematologists who experience added cognitive load to check a tool intended to help them. While it is rather complicated to find out what made a deep learning algorithm make a mistake while classifying some cell morphology or flow MFC gating, it is important to note that, since currently AI is regarded as a “decision support tool,” the ultimate professional and legal responsibility lies with a human physician who approved the test result. To reduce these risks, it should be noted that clinical workflow never allows AI to work independently. Indeed, AI becomes a pre-screening tool or a “second pair of eyes.” Also, all abnormal cells detected by AI and the complex flow cytometry gating strategy are double-checked and confirmed by a hematopathologist. As such, given the potential effects on the patient, the regulatory system requires that, in accordance with ICC guidelines, all machine decisions be verified by a hematopathologist. Through this approach, the machine will act as an aid in pre-screening or triggering patients, with the physician making the ultimate decision. In this context, the ICC, as well as other international activities, namely the International Council for Standardization in Hematology (ICSH), require the use of AI exclusively as an auxiliary element following the Human-in-the-Loop concept. Hence, the AI algorithm completes the procedure and normalizes data interpretation, while the hematopathologist makes the final diagnosis. Four main principles for using AI technology in modern hematopathology at the international level include multimodal integration, such as the MICM (morphology, immunophenotyping, cytogenetics, and molecular diagnostics) framework. In this way, modern DL systems should be compatible with international diagnostic criteria via the multimodal analysis of all MICM parameters. From this standpoint, the focus on genomic variants and the linkage of somatic mutations with cellular morphology will improve risk stratification. Second, workflow standardization and infrastructure improvements are needed to reduce inter-observer variability and increase laboratory efficiency; this need is stated in the consensus statements. In this regard, technical interoperability presupposes the adoption of open standards to integrate third-party AI modules with Laboratory Information Systems and digital pathology scanners. Simultaneously, pre-analytical automation will offer an opportunity to standardize the quality of whole-slide imaging scans and to avoid artifacts generated by variable staining and thickness. Finally, clinical decision support uses AI to pre-draft the diagnostic report and provide differential diagnoses from electronic health records, thereby reducing cognitive load. In contrast, clinical governance and risk management are the third pillar. Here, consensus committee recommendations require implementing a strict risk management policy due to the limitations of deep learning technology, including the “Black Box” Problem, bias, edge cases, and Ethical Frameworks. In this respect, consensus committee recommendations imply a strict risk management strategy to address the limitations of deep learning technology, such as the “Black Box” Problem, bias, edge cases, and Ethical Frameworks. According to the recommendations, hematopathologists need to be especially careful about a model’s lack of explainability, as it may overlook rare, subtle morphological features. Furthermore, AI is often inefficient at recognizing ambiguous stages of cell maturity (e.g., atypical promyelocytes) and rare diseases. Human validation is mandatory for any outlier and underrepresented cases in the training dataset. However, the implementation should adhere to international AI guidelines regarding fairness, universality, traceability, usability, robustness, and explainability [9]. Finally, the fourth pillar deals with education and curriculum updates. In this regard, the changeover to AI-enabled diagnosis calls for a complete revamp of the educational process, in which specialized training in data science for healthcare and ethics/legal litigation will be integrated into pathology residencies, with critical evaluation and auditing of AI outputs [67,68].

## 5. Prognosis and Risk Assessment in AML

AML is highly diverse, so patients with similar clinical features may experience very different outcomes. Therefore, prognostic prediction and risk assessment are essential for selecting the most appropriate clinical management. The current risk stratification and management guidance systems, such as the European LeukemiaNet (ELN) and the National Comprehensive Cancer Network, are designed to assess genetic risk using standard cytogenetics and molecular biomarkers to assign patients to favorable, intermediate, or adverse risk profiles [33,34]. These approaches play an important role in decision-making, yet their linear design hampers their ability to recognize interactions among many factors, such as somatic mutations and patient characteristics [58]. In this regard, AI models enable the integration of multiple disease characteristics, including cytogenetics, mutation profiles, clinical variables, laboratory values, and treatment history [37]. Thus, these systems aim to predict overall survival (OS), relapse risk, treatment response, and transplant outcomes [37]. Systematic review papers show that AI-based prognostic models tend to outperform classical risk scoring, but external validation poses a great challenge before wide-scale implementation into clinical practice [37]. The emergence of multi-omics (genomics, epigenomics, transcriptomics, proteomics, and metabolomics) approaches has contributed to a comprehensive view of the mechanisms underlying leukemia development, its heterogeneity, and drug targets [37,58,59]. Thanks to the application of high-throughput sequencing, mass spectrometry, and computational technologies, new biomarkers of AML, patterns of clonal evolution, and microenvironmental interactions have been found. AI is especially helpful for processing multidimensional data and discovering new patterns and drug vulnerabilities that might go unnoticed by classical statistical analysis methods [58]. Undoubtedly, one of the most promising AI applications is predicting patients’ response to specific treatments, such as Venetoclax-based combinations, Hypomethylating agents (HMAs), targeted inhibitors, immunotherapy, hematopoietic cell transplantation (HCT), and Chimeric Antigen Receptor T-cell (CAR-T) [5,8,38,69,70,71,72]. In this respect, the ML algorithm might analyze patients’ molecular profiles and treatment histories to estimate response probabilities [7,37,73,74,75,76]. In this connection, a newly published paper confirmed the applicability of the ELN 2022 criteria for the classification of AML patients by genetic risk and found groups of patients sharing specific cytogenetics and somatic mutations associated with similar survival rates according to three treatment regimens, including IC, HMAs, and the latter agents associated with venetoclax [73]. There were no differences in OS rates among the three risk categories defined by the ELN 2022 criteria. Using AI-based genomic analysis, researchers were able to distinguish nine groups of AML patients with differential survival across treatment options. Based on the provided information, the authors argue that the currently used ELN criteria are inadequate for genetic risk assessment in AML patients over 60 years of age and emphasize the need for a more comprehensive genetic risk classification that incorporates coexisting somatic mutations [73]. Nevertheless, ML models use retrospective data and cannot yet provide prospective validation. Though highly precise, convolutional neural network algorithms do not allow for easy explanation to a physician of the grounds for classifying a particular patient as high risk [74]. Therefore, even promising technologies like AI require further improvement [75]. Once more, AI-driven DL pipelines can monitor MRD during treatment, enabling early detection of resistance or relapse and allowing adjustments to therapeutic approaches before clinical deterioration [76]. In this light, AI-assisted analysis of MFC, sequencing data, and digital pathology may improve the sensitivity and reproducibility of MRD detection while reducing interpretation time [42]. Again, convolutional neural networks are combining blood cell counts with biochemical spectroscopy to detect early relapse or find high-risk patients, achieving up to 98% accuracy [76,77,78,79]. Table 4 highlights the main differences between traditional and computational approaches in AML prognostication and risk assessment [33,34,37]. Therefore, the AI-based methods allow the incorporation of all patient information, making predictions much more precise than traditional methods. As a result, the process becomes personalized, enabling the development of more effective monitoring and intervention methods. Soon, a compromise will likely be reached as physicians use both their experience and the structured approach of existing recommendations [80].

## 6. AI-Enabled Patient Evaluation and Treatment Selection

AI-enabled personalized assessment of patients with AML dramatically speeds up diagnosis, improves risk stratification, and helps select personalized treatment. By incorporating various biomedical inputs, AI standardizes medical processes and improves accuracy beyond what manual control can ensure. The AI-enabled evaluation process combines a patient’s genomic markers, biological age, and the BM environment. Unlike traditional approaches that rely on protocol-based treatment, DL algorithms use unique parameters to provide the best fit and recommend an optimal treatment course that minimizes toxicity while accurately identifying candidates for HCT [37]. Traditional methods treat each genetic alteration as an independent, static parameter. ML-based algorithms consider mutations in a complex interconnected network of factors. Thus, ML models apply the VAF concept to determine which mutation is ancestral (dominant) and which is subclonal, thereby enabling accurate predictions of the efficacy of targeted therapy. For example, if there is suspicion that an *FLT3* inhibitor (like gilteritinib) is likely to cut off only a minor part of a tree rather than kill all its branches, the AI would suggest alternative drug combinations [37,78]. Moreover, an important element ML uses to develop its algorithms is the understanding that certain genetic alterations occur in sequence. In this regard, when an *NPM1* mutation is associated with *DNMT3A* and *FLT3-ITD* alterations, the algorithm recalculates the patient profile as “high risk” rather than “favorable,” thereby recommending that the patient undergo HCT after the first remission [81]. When a patient has a complex karyotype and Tumor Protein 53 (*TP53)* mutations, which are typically associated with bad IC results, AML therapeutic algorithms would suggest participation in clinical trials testing new therapeutic agents or using non-intensive strategies, including HMAs with venetoclax therapy, to minimize futile toxicity. Another aspect of individual assessment provided by AI-based algorithms is a numerical estimate of a patient’s biological age, which replaces a traditional subjective approach. For example, Automated Comorbidity Indexing models are used to evaluate the probability of treatment-related mortality [80]. They automatically scan a patient’s electronic health record to assess their HCT-Comorbidity Index (HCT-CI) [81]. This index assigns weights to certain underlying cardiac, pulmonary, or renal conditions and thus helps estimate the risk of IC-related mortality. Finally, ML algorithms analyze MFC data and protein expression patterns (CD33, CD123, or CLL-1) to determine whether a patient could receive help from antibody-drug conjugate therapy (gemtuzumab ozogamicin) or adoptive cell transfer (CAR-T cell therapy) [72,82].

## 7. Traditional Fitness Criteria Assessment vs. Metrics Using AI in AML

One of the most important conceptual insights from the study of AML is that fitness is not fixed at the time of diagnosis but is dynamic and continually evolving throughout the patient’s journey [30,82,83]. Therefore, traditional milestone concepts of fitness in AML and their reviews are reported in Table 5 [84,85,86,87]. In addition to traditional criteria, valuable efforts to assess the objective patient’s fitness have been reported [87,88]. However, unprecedented advances in this field are driven by ML approaches that analyze complex, nonlinear patient data from clinical, laboratory, genetic, and real-time digital sources to predict patients’ ability to tolerate therapy. As shown in Table 6, these AI-based criteria compare with traditional fitness measures and can capture changes in fitness states, providing a deeper understanding of this complex biological process [58,81,89,90,91,92,93]. Moreover, supervised ML techniques enable the identification of prognosis-related patterns from electronic health records at admission, thereby ending the subjectivity of manual performance status assessments [90,93]. Further advances in this field include Wearable Technologies and Digital Biomarkers of Frailty, which could play a fundamental role in the modern management of AML. Indeed, the convergence of AI with wearable device technology offers a new paradigm for remote patient monitoring beyond the clinic [91,92]. Continuous sensor data, including average heart rate, oxygen saturation, skin temperature, and sleep habits, can be accessed remotely in real time to provide a “snapshot” of a patient’s baseline behavior. In addition, wearable-derived physical activity metrics have shown high clinical value. Higher activity levels during recovery from cancer-related stressors are associated with fewer readmissions. Specifically, a higher step count per minute is linked to a reduced risk of falls and improved OS [90,91]. The implementation of AI analytical tools will enhance clinical outcomes in the remote monitoring of patients’ conditions [92,93]. Notably, remote home-based electronic monitoring significantly improves QoL [94,95]. Hence, AI may provide objective evidence of functional status by serving as a “third party” between the patient’s feelings and the physician’s interpretation of the patient’s complaints.

## 8. Implementation of Electronic QoL Assessment and Potential AI Application in AML

AML is one of the most aggressive hematological malignancies, characterized by marked biological heterogeneity and requiring prompt, intensive treatment, which, together with treatment-related toxicities—including cytopenias, life-threatening sepsis, and severe mucositis—can cause a sudden disruption to patients’ lives and lead to substantial physical and psychological distress [8,98,99,100]. An AML diagnosis, accompanied by prompt treatment, shocks patients to such an extent that they feel like being “abducted”. Traditionally, progression-free survival (PFS) and OS are considered key indicators for the treatment of oncological patients. Nonetheless, there is a considerable paradigm shift occurring in clinical practices towards more patient-oriented medical care [101]. Intensive AML treatment makes patients focus primarily on QoL, reduction in symptom burden, hospitalization duration, and costs, rather than increasing survival rates. Because AML treatment is highly intensive, it negatively affects patients’ health and functional capacities [101]. To address the profound psychological distress and sudden trauma of an AML diagnosis, digital therapeutics have emerged as highly scalable, accessible solutions to bridge the gap in clinical supportive care [101]. In addition, palliative care interventions have proven highly effective at mitigating anxiety, depression, and physical symptom burden in leukemia patients [8]. However, healthcare schools face a severe shortage of specialized supportive care clinicians. In response, researchers have translated the active, evidence-based components of clinical palliative interventions into self-administered digital platforms [8,94,95,102]. Standard clinical practice relies heavily on periodic, subjective clinical assessments and laboratory testing. Nevertheless, laboratory data alone do not capture how a patient feels and functions in everyday life. Indeed, Patient-Reported Outcomes (PROs) provide unique clinically relevant information [103]. In this regard, the electronic-PROs (ePROs) modality may address barriers to completion [104]. An example of the use of ePRO to assess patient health status is seen in studies aimed at personalizing transfusion support [105]. In this regard, the WearAble Technology for Collecting Health data in people who are Transfused (WATCH) clinical study was conducted at the University of Oxford (NCT07511829, https://clinicaltrials.gov/study/NCT07511829: accessed 31 May 2026) in adult patients receiving treatment for MDS and AML, using step count and sleep quality indicators, together with hematological indices, to train an AI algorithm that enables the provision of personalized supportive care. Blood transfusions will thus be used only when functional capabilities decline, thereby improving physical QoL and minimizing potential side effects according to the study hypothesis that classical fixed transfusion thresholds do not consistently correlate with the level of functional impairment (NCT07511829). Again, the use of ePROs is further supported by literature reviews, such as the one conducted by Warnecke and colleagues, which found that electronic methods enhance the assessment of physical and mental conditions that healthcare providers may otherwise underestimate [106].

## 9. AI-Assisted Clinical Research in AML

Given its biological and clinical complexities, AML is one of the areas in which AI has been successfully applied across various stages of clinical investigation, from diagnosis to the course of disease. In such a setting, AI-based algorithms for AML diagnosis have demonstrated that automated optical image processing can facilitate clinical decision-making in interpreting AML status from microscopic images of PB and BM [40,45,65]. Moreover, traditional AML prognostic models are based on clinical and genetic factors [33,34,35]. Nevertheless, due to numerous complications and the disease’s dynamism, ML techniques can be considered quite helpful for improving AML prognosis [37]. In this regard, an innovative predictive AML model has been developed using 13 programmed cell death pathways, including apoptosis, ferroptosis, pyroptosis, and autophagy, and employs ML to generate predictive models from 73 combinations of 10 different algorithms [107]. However, the most widely explored aspect regarding AI and AML treatment is predicting drug responsiveness [5,8,18,36,37,54,70]. Indeed, given the disease’s pronounced heterogeneity and the individual patient’s fitness, it is crucial to determine the drug regimen for each patient [11,38]. In this regard, the NetAML platform has recently developed 87 drug-sensitivity prediction models for 87 clinical drugs, using RNA sequencing (RNA-seq)-based ex vivo drug-response data from 520 AML patients. It employs network-based analysis and ML algorithms to identify biologically meaningful signatures of drug resistance and sensitivity [108]. Another important example is a work by the BeatAML consortium, which has recently combined genomic, transcriptomic, and ex vivo drug-sensitivity profiling data from 672 AML patients, using ML algorithms to develop highly predictive models, representing one of the greatest and most comprehensive efforts in the multi-omics space [109]. Additionally, the use of AI tools in AML trials has also been proven [22,42,91,110]. AI algorithms are involved in stratifying, recruiting, and monitoring treatment compliance in AML patients. They can analyze study protocols to identify issues and inefficiencies and forecast clinical trial outcomes. The value of AI models in AML is immense, paving the way for the continued evolution of these technologies in such research settings. In this regard, generative AI models have become a source of discoveries. For instance, AI generative algorithms can generate synthetic patient populations and “digital twins” of individual patients, enabling the simulation of disease progression and treatment dynamics [7,110,111,112]. These digital twins allow researchers to test personalized sequences of non-cross-resistant therapies in a simulated environment before administering them to the patient [7]. Dynamic disease progression simulations of digital twins can indicate that such adaptations may increase survival rates by predicting the kinetics of drug resistance before clinical diagnosis [113]. This model is particularly useful in rare cases of AML, such as BCR: ABL1 and inv(3)/t(3;3), for which few patients are enrolled in clinical studies [114]. Therefore, it plays a valuable role in helping AML specialists improve clinical trials to develop new treatments. Still, it is necessary to conduct rigorous ethical, regulatory, and methodological evaluations of synthesized data before their application in clinical practice. Nevertheless, the number of challenges AML specialists face continues to grow. Some of them are related to selection bias in training datasets, limited prospective validation studies, poor data quality, and lack of regulation [115,116,117].

## 10. Ethical, Legal, and Implementation Issues Related to the Use of AI in AML

Although there is great potential for using AI in the management of AML, many issues need to be addressed first. Apart from technological issues, there are also ethical, legal, and implementation issues related to the use of AI in medicine [114]. First, DL techniques are associated with an issue known as “black box”. In other words, there is no clear explanation of how an algorithm makes a particular decision, and this opacity violates major ethical principles, such as autonomy, because clinicians cannot explain the rationale behind an algorithm-driven suggestion to their patients [116,117,118].

## 11. Algorithmic Bias and Health Justice

AI systems are highly sensitive to the quality of the data used in their development, and that data may be biased or representative only of a limited population [114]. In oncology, where structural racism and socioeconomic determinants of health already change outcomes, prejudicial algorithms are a serious threat to patients’ equity and personal privacy [117]. In this regard, AI-assisted learning approaches can enable training models on multi-institutional datasets without exposing patient information, thereby supporting confidentiality and addressing privacy concerns [9]. Lastly, strict data provenance and data quality guidelines must be put in place to help in making AI systems fair and reliable, regardless of the population subgroup [118].

## 12. Regulatory Oversight and Medico-Legal Liability

Transitioning from the development stage to implementation requires a proper governance structure. Medical devices using AI have received clearance from regulatory agencies such as the FDA. Nevertheless, existing regulations may not apply, as many are tailored to non-adaptive software [115]. Adaptive learning models pose challenges in such cases and require post-market surveillance to ensure continued effectiveness and safety [115]. More importantly, it is unclear where liability should fall if mistakes occur [119].

## 13. Conclusions and Directions for the Future

Although the field of hematology–oncology is rapidly shifting from single-modality proof-of-concept studies to multimodal AI solutions, present-day technologies are used as specialized “co-pilots” rather than absolute deciders. While previous technologies were based on centralized databases, the latest models use federated architecture and multimodal data collection, including MFC and genomic data. However, currently, no single model in regular clinical practice can, on its own, take a raw morphological image, MFC results, multi-omics data, and information on the patient’s fitness to define a treatment regimen. Consequently, AI is not deciding what the final treatment regimen will be. Many organizations responsible for clinical trials, research groups, and large international organizations, such as those in Europe and North America, are actively developing data exchange systems to build multimodal AI models capable of detecting AML and assisting with its management in daily clinical practice [6,78]. However, the use of AI in medical decision-making has changed our understanding of AML and will profoundly impact our clinical practice. For example, using age-based criteria versus a multidimensional approach to fitness will have different implications for patient care. First, AI removes subjectivity, preventing undertreatment and overtreatment [4]. Also, using electronic health record analytics and wearable technology, AI might enable dynamic monitoring of patients’ fitness during therapy rather than at admission [90,91,92,93,95,102]. Nevertheless, challenges exist in implementing AI-based algorithms. Randomized trials are essential to figure out whether AI-based therapeutic strategies yield better survival rates and QoL than traditional clinician-based strategies [37]. Standardization is another vital aspect to consider for reporting and validating demographic bias, among other things [37,54]. Ultimately, AI should be viewed not as a replacement for clinical expertise but as a sophisticated virtual assistant that empowers the hematologist [78,79]. By processing vast amounts of literature, genomic data, and real-time patient history, AI enables the realization of true precision medicine in AML: delivering the right therapy at the right intensity to the right patient at exactly the right time [7,13,18,37]. Additionally, AI is revolutionizing the field of AML research through the development of multimodal early-screening methods, single-cell technologies to discover new CAR-T targets, and prediction models for patient survival. Using DL, ML, and natural language processing, scientists have been rapidly speeding up the discovery process in precision medicine for AML. Finally, in an ever-changing world of AML treatment, AI can help restore humanity in medicine by automating the cognitive functions doctors perform routinely and enabling them to spend time with their patients rather than on administrative tasks. AI will handle all documentation, allowing doctors to focus on providing personalized, caring healthcare to patients. For example, AI-based medical scribes and ambient listening technology can automatically document doctor-patient visits and upload all relevant information to electronic health record systems, allowing doctors to maintain eye contact with their patients. Also, AI can automatically translate medical terminology into plain language for patients. Thus, patients become more involved in decision-making as they begin to understand how their illness affects their bodies and how it can be treated. Furthermore, because of its ability to analyze large datasets, AI can detect risk factors specific to a particular patient, including their genetics and lifestyle [120,121]. This way, specialists can develop a truly personalized and holistic treatment regimen that addresses not only the problem but also its potential causes. As usual, AI-powered technologies can reduce weaknesses inherent in humans, such as their inability to process vast amounts of information at once and to avoid burnout [41,78]. Nevertheless, clinical intuition, empathy, and ethics are unique human qualities that no machine will replace [122].

## Figures and Tables

**Table 1:** The Taxonomy of Intelligent Systems [7,22].

Attribute	AI	ML	DL
Scope and Definition	Broadest concept: creating systems that simulate human intelligence, reasoning, and feeling.	A subset of AI that develops systems capable of learning from data to make decisions autonomously, without explicit programming.	A specialized subset of ML using multi-layered ANNs to learn complex patterns from raw data.
Primary Goal	To imitate or reproduce human intelligence in machines across different fields.	To enable machines to learn from data to perform specific tasks with increasing accuracy.	To achieve high accuracy in complex tasks (unstructured data) by automatically learning features.
Data Requirements	Highly variable; rule-based systems may require minimal data, while others need massive sets.	Requires significant amounts of structured or labeled data for effective training.	Complex network training is performed on large datasets with millions of samples.
Hardware Requirements	Depends on whether it is simple or complex artificial intelligence. Simple AI requires normal computing, but complex AI needs more power.	Normal CPUs can handle it, although complex models require GPU support.	It usually demands high-performance computing resources, especially GPUs or TPUs, to handle parallel processing.
Learning Approach	Employs diverse techniques including logic, rule-based systems, search algorithms, and optimization.	Includes linear regression, SVMs, and decision trees, among other learning algorithms.	Uses multi-layered neural networks inspired by biological brain structures with many parameters.
Feature Engineering	Traditionally, it requires human intervention to define rules and symbolic representations.	Often relies on human intervention to categorize data and highlight specific attributes.	Automatically extracts hierarchical features from data, reducing the need for manual engineering.

AI: Artificial Intelligence; ML: Machine Learning; DL: Deep Learning; ANNs: Artificial Neural Networks; CPUs: Central Processing Units; GPU: Graphics Processing Unit; TPUs: Tensor Processing Units; SVMs: Support Vector Machines.

**Table 2:** Morphological features used by AI models to predict genomic signatures.

Mutation/Fusion	Morphological Hallmark	AI Feature Extraction Focus	Clinical Utility
*NPM1*	Cup-shaped nuclear morphology.	Nuclear indentation and cytoplasmic texture.	Rapid screening for favorable risk.
*t(15;17)* (APL)	Atypical promyelocytes, Auer rods.	Granularity and nuclear shape.	Immediate initiation of ATRA.
*FLT3*	High blast count, non-specific texture.	Cellularity and fine chromatin patterns.	Early identification of high-risk cases.
*t(8;21)*	Large blasts, perinuclear halos.	Cytoplasmic clearing and cell size.	Prognostic stratification.
*RUNX1::RUNX1T1*	Large-to-medium blasts with irregular, indented nuclei.	The cytoplasm is abundant and light blue, with purplish-red granules. Near the nucleus, a clear Hof mark the Golgi zone. Long Auer rods are common, sometimes forming larger “pseudo-Chediak-Higashi” granules.	Prognostic stratification.

AL: artificial intelligence; *NPM1*: nucleophosmin-1; APL: acute promyelocytic leukemia; ATRA: all-trans retinoic acid; *FLT3*: FMS-like tyrosine kinase 3; *RUNX1::RUNX1T1*: **R**unt-**R**elated Transcription Factor 1/**RUNX1 P**artner **T**ranscriptional Co-Repressor 1.

**Table 3:** AI-assisted MFC [50,51,55].

Diagnostic Challenge	AI Performance (AUROC)	Biological Mechanism
AML vs. ALL Differentiation	0.965	Surface marker expression patterns
Presence of AL	0.961	Detection of aberrant blast populations
t(15;17) Prediction	0.929	APL-specific immunophenotype
NPM1 Variant Prediction	0.807	Myeloid lineage correlations
t(8;21) Prediction	0.814	RUNX1::RUNX1T1 phenotype

AI: artificial intelligence; MFC: multiparametric flow cytometry; AUROC: Area Under the Receiver Operating Characteristic; AML: acute myeloid leukemia; ALL: acute lymphoblastic leukemia; AL: acute leukemia; APL: acute promyelocytic leukemia; NPM1: nucleophosmin-1; RUNX1::RUNX1T1: Runt-Related Transcription Factor 1/RUNX1 Partner Transcriptional Co-Repressor 1.

**Table 4:** Comparison between traditional and AI-assisted approaches for Prognostication and Risk Assessment in AML [37].

Feature	Traditional Methods (e.g., ELN and NCCN Guidelines) [33,34]	AI-Assisted Prognostication
Primary Data Source	Standardized, low-dimensional (Cytogenetics, selected molecular markers, age).	High-dimensional, multi-omic (Genomics, transcriptomics, proteomics, imaging, EHR).
Logic/Approach	Rule-based, hierarchical classification systems (favorable/intermediate/adverse).	Data-driven, pattern recognition (Machine/Deep Learning).
Dynamic Ability	Static; requires manual updates to guidelines as knowledge evolves.	Dynamic; adapts and improves performance as it processes new, real-time data.
Pattern Recognition	Limited to predefined, well-known biomarker combinations.	Capable of uncovering non-linear, complex, and novel prognostic signatures.
Interpretability	High, adhering to proven medical logic and clear clinical rules.	Variable; often meets the “black box” issue, needing Explainable AI techniques.
Speed/Scale	Requires significant time; depends on manual pathology and expert review.	Almost instant; handles large datasets at scale with high levels of automation.
Goal	Grouping patients into broad risk categories for standard protocols.	Precision medicine: predicting individual patient outcomes (e.g., relapse, survival time).

AI: artificial intelligence; ELN: European Leukemia Net; NCCN: National Comprehensive Cancer Network; EHR: electronic health records.

**Table 5:** Traditional criteria and tools of patients’ fitness in AML [30,82,83,84,85,86].

Assessment Tool	Evaluated Domains and Key Parameters	Clinical Scoring Thresholds	Primary Diagnostic Role and Limitations
ECOG Performance Status	Physical activity, self-care capacity, and daily functional limitations.	Scale from 0 (fully active) to 5 (completely disabled).	Standard tool for IC eligibility; highly subjective and easily confounded by reversible, leukemia-induced acute symptoms.
HCT-CI (Sorror Index)	Categories of objective organ dysfunction, including cardiac, pulmonary, renal, and hepatic impairments.	Point-based sum score indicates a high comorbidity burden.	Standardized predictor of HCT-related and NRM requires structured laboratory and functional test inputs.
Ferrara Criteria (SIE/SIES/GITMO)	Consensus-based host organ function thresholds, active infections, cognitive status, and age.	Binary classification (unfit if any conceptual or operational criterion is met).	Identifies patients who are ineligible for IC; lacks multidimensional measures of functional reserve and frailty.
Geriatric 8 (G8) Score	Nutrition, weight loss, BMI, mobility, neuropsychological problems, polypharmacy, and self-rated health.	Scale of score defining frailty or unfitness.	A rapid geriatric screening tool requires manual clinical administration and is time-consuming in routine practice.

ECOG: Eastern Cooperative Oncology Group; IC: intensive chemotherapy, HCT-CI: hematopoietic cell transplantation-comorbidity index; HCT: hematopoietic cell transplantation; NRM: non-relapse mortality; BMI: body mass index.

**Table 6:** Complete Overview of Fitness Modalities.

Comparison Dimensions	Traditional Criteria	AI-Assisted and ML Models
Core Evaluation Paradigm	Static clinician scales, comorbidity indexes [30,83].	Automated, data-driven prognostic modeling integrated with real-time digital biomarker analysis [37].
Data Modalities	Includes chronological age, static lab values, baseline organ function tests, and clinician-rated PS [2,83].	Multi-omics, high-frequency actigraphy, sleep architecture, facial images, and clinical text [58,96].
Temporal Resolution	A single, separate pre-treatment baseline measurement taken at the time of initial diagnosis [82].	Continuous, real-time longitudinal monitoring during daily activities and active therapy in free-living conditions [96].
Inter-Observer Subjectivity	Despite its susceptibility to clinician bias and diagnostic confounding, the HCT-CI’s reproducibility improves with training programs [83].	Algorithmic assessment reduces subjective bias but requires cross-institutional model validation to avoid it [37,79,84].
Confounding by Disease Burden	High, acute, and reversible leukemia symptoms such as anemia, infection, and leukostasis can resemble chronic physiological frailty [83].	Low; models adjust for leukemia burden by including cytogenetics, mutational signatures, and metabolic cofactors [96].
Type and Accuracy	Categorical groups like fit versus unfit, or low, intermediate, or high risk [86].	Continuous, individualized probability curves for OS, CR, relapse, and NRM [96].
Representative Models	ECOG PS, HCT-CI comorbidity index, Ferrara Criteria, G8, and the AML-CM [8,83].	Sanger AML Knowledge Bank, ML Early Death Classifiers, Garmin Venu SQ Smartwatch, FaceAge, TrialGPT [81,97].
Primary Clinical Benefit	This approach is internationally recognized, widely accepted, and does not need complex infrastructure or specialized software [83].	Highly personalized, this system adapts dynamically to functional changes and detects subclinical toxicities [96].
Implementation Barriers	Manual clinical assessments are time-consuming and do not benefit from continuous validation via multicenter trials [83].	Challenges include EHR integration, strict data privacy laws, “black box” explainability, and validation across different cohorts [37,97].
Role in the Clinical Pathway	Guides the initial treatment choice at diagnosis, determining whether to use IC or a less intensive targeted therapy [2,8,83].	Offers ongoing decision support, guiding post-remission HCT, remote monitoring, and clinical trial matching [97].

AI: artificial intelligence; ML: machine learning; PS: performance status; HCT-CI: hematopoietic cell transplantation-comorbidity index; OS: overall survival; NRM: non-relapse mortality; ECOG: Eastern Cooperative Oncology Group; AML-CM: acute myeloid leukemia-composite model; HER: electronic health records; IC: intensive chemotherapy.

## Data Availability

The original contributions presented in this study are included in the article. Further inquiries can be directed to the corresponding author.

## References

[1] Shimony S., Stahl M., Stone R.M. (2025). Acute Myeloid Leukemia: 2025 Update on Diagnosis, Risk-Stratification, and Management. Am. J. Hematol..

[2] Jeurkar C., King L., Baek D., Wilde L., Keiffer G., Kasner M. (2026). Management of Acute Myeloid Leukemia: A Review. Cancers.

[3] Sobas M.A., Turki A.T., Ramiro A.V., Hernández-Sánchez A., Elicegui J.M., González T., Melchor R.A., Abáigar M., Tur L., Dall’Olio D. (2025). Outcomes with intensive treatment for acute myeloid leukemia: An analysis of two decades of data from the HARMONY Alliance. Haematologica.

[4] Al-Shibly R., Benkhadra M., Yassin M.A. (2026). Transforming Acute Myeloid Leukemia Care Through Precision Medicine: Integrating Genomic Profiling, Measurable Residual Disease, and Targeted Therapies. Oncology.

[5] Ko K.H., Gelfer R., Wheat J.C., Cai S.F. (2026). Targeted Therapy in Acute Myeloid Leukemia: Current Approaches and Novel Directions. J. Pers. Med..

[6] Li Z., Zhao J., Huang L., Chen X., Wei J., Tian W. (2026). Artificial intelligence reshaping the paradigm of hematologic malignancy diagnosis and treatment: From static assessment to dynamic precision management. Ann. Hematol..

[7] Al-Nusair J., Lanino L., Durmaz A., Della Porta M.G., Zeidan A.M., Kewan T. (2025). Artificial intelligence in myeloid malignancies: Clinical applications of machine learning in myelodysplastic syndromes and acute myeloid Leukemia. Blood Rev..

[8] Niscola P., Gianfelici V., Giovannini M., Mazzone C., Del Principe M.I. (2026). Evolving Management Approaches Toward Personalized Therapy in Acute Myeloid Leukemia: A Narrative Review. J. Pers. Med..

[9] Ansarian M.A., Fatahichegeni M., Xu R., Chen Y., Wang X., Ren J., Liu H. (2025). Emerging Artificial Intelligence Technologies for Risk Assessment and Management in Acute Myeloid Leukemia: A Review. JAMA Oncol..

[10] Achir A., Debbarh I., Zoubir N., Battas I., Medromi H., Moutaouakkil F. (2025). Advances in Leukemia detection and classification: A Systematic review of AI and image processing techniques. F1000Research.

[11] Ali A., Kausar M.A., Anwar S., Khan F.H., Elagib H.M., Shahien M.M., Alshabrmi F.M. (2026). The expanding role of artificial intelligence in personalized medicine: From innovation to individualized care. Front. Med..

[12] Gimeno M., San José-Enériz E., Villar S., Agirre X., Prosper F., Rubio A., Carazo F. (2022). Explainable artificial intelligence for precision medicine in acute myeloid leukemia. Front. Immunol..

[13] Haferlach T., Eckardt J.N., Walter W., Maschek S., Kather J.N., Pohlkamp C., Middeke J.M. (2025). AML diagnostics in the 21st century: Use of AI. Semin. Hematol..

[14] Wang S.X., Huang Z.F., Li J., Wu Y., Du J., Li T. (2024). Optimization of diagnosis and treatment of hematological diseases via artificial intelligence. Front. Med..

[15] Wang J. (2024). Deep Learning in Hematology: From Molecules to Patients. Clin. Hematol. Int..

[16] Gedefaw L., Liu C.F., Ip R.K.L., Tse H.F., Yeung M.H.Y., Yip S.P., Huang C.L. (2023). Artificial Intelligence-Assisted Diagnostic Cytology and Genomic Testing for Hematologic Disorders. Cells.

[17] Găman M.A., Dugăeşescu M., Popescu D.C. (2025). Applications of Artificial Intelligence in Acute Promyelocytic Leukemia: An Avenue of Opportunities? A Systematic Review. J. Clin. Med..

[18] Abdul Rasool Hassan B., Mohammed A.H., Hallit S., Malaeb D., Hosseini H. (2025). Exploring the role of artificial intelligence in chemotherapy development, cancer diagnosis, and treatment: Present achievements and future outlook. Front. Oncol..

[19] Asadov C., Shirinova A., Alimirzoyeva Z., Hasanova A. (2026). Digital Medicine and Artificial Intelligence in Chronic Myeloid Leukemia: Current Applications, Challenges, and Future Directions. Cureus.

[20] Liao H., Zhang F., Chen F., Li Y., Sun Y., Sloboda D.D., Zheng Q., Ying B., Hu T. (2025). Application of artificial intelligence in laboratory hematology: Advances, challenges, and prospects. Acta Pharm. Sin. B.

[21] Xiang J., Shi M., Fiala M.A., Gao F., Rettig M.P., Uy G.L., Schroeder M.A., Weilbaecher K.N., Stockerl-Goldstein K.E., Mollah S. (2022). Machine-learning-based scoring models for predicting hematopoietic stem cell mobilization in allogeneic donors. Blood Adv..

[22] Sarker I.H. (2021). Deep Learning: A Comprehensive Overview on Techniques, Taxonomy, Applications and Research Directions. SN Comput. Sci..

[23] Turki A.T., Fan Y., Hernández-Sánchez A., Silva W., Fleming S., Yalcin K., Van Elssen C.H.M.J., Madanat Y., Karasek M., Aljurf M. (2026). International testing and refinement of AI algorithms predicting acute leukemia subtypes from routine laboratory data. Nat. Commun..

[24] Alhajahjeh A., Nazha A. (2024). Unlocking the Potential of Artificial Intelligence in Acute Myeloid Leukemia and Myelodysplastic Syndromes. Curr. Hematol. Malig. Rep..

[25] Bao J., Freund O., Sund L., Du W. (2026). Inside the Battle Against Acute Myeloid Leukemia: Biology, Breakthroughs, and Hope. Cells.

[26] Kantarjian H., Chifotides H.T., Haddad F.G., Jabbour E., Jain N., Kadia T., Ravandi F., Welch M.A., Wierda W., Freireich E.J. (2026). The Care and Cure of the Leukemias in 2026. Am. J. Hematol..

[27] Khoury J.D., Solary E., Abla O., Akkari Y., Alaggio R., Apperley J.F., Bejar R., Berti E., Busque L., Chan J.K.C. (2022). The 5th edition of the World Health Organization Classification of Haematolymphoid Tumours: Myeloid and Histiocytic/Dendritic Neoplasms. Leukemia.

[28] Arber D.A., Orazi A., Hasserjian R.P., Borowitz M.J., Calvo K.R., Kvasnicka H.M., Wang S.A., Bagg A., Barbui T., Branford S. (2022). International Consensus Classification of Myeloid Neoplasms and Acute Leukemias: Integrating morphologic, clinical, and genomic data. Blood.

[29] Koorse Germans S., Weinberg O.K. (2026). Navigating diagnostic shifts: A comparative review of the WHO 5th edition and ICC 2022 classifications of myeloid neoplasms. Hum. Pathol..

[30] Venditti A., Palmieri R., Maurillo L., Röllig C., Wierzbowska A., de Leeuw D., Efficace F., Curti A., Ngai L.L., Tettero J. (2025). Fitness assessment in acute myeloid leukemia: Recommendations from an expert panel on behalf of the European LeukemiaNet. Blood Adv..

[31] Cloos J., Valk P.J.M., Thiede C., Döhner K., Roboz G.J., Wood B.L., Walter R.B., Wang S.A., Wierzbowska A., Wei A.H. (2026). 2025 Update on MRD in Acute Myeloid Leukemia: A Consensus Document from the ELN-DAVID MRD Working Party. Blood.

[32] Niscola P., Piccioni D., Giovannini M. (2025). Palliative care interventions and their early integration in the management of older adults with acute myeloid leukemia: A narrative review. Ann. Palliat. Med..

[33] Döhner H., Wei A.H., Appelbaum F.R., Craddock C., DiNardo C.D., Dombret H., Ebert B.L., Fenaux P., Godley L.A., Hasserjian R.P. (2022). Diagnosis and management of AML in adults: 2022 recommendations from an international expert panel on behalf of the ELN. Blood.

[34] Pollyea D.A., Altman J.K., Assi R., Bixby D., Fathi A.T., Foran J.M., Gojo I., Hall A.C., Jonas B.A., Kishtagari A. (2023). Acute Myeloid Leukemia Version 32023, NCCNClinical Practice Guidelines in Oncology. J. Natl. Compr. Cancer Netw..

[35] Ruhnke L., Bill M., Zukunft S., Eckardt J.N., Schäfer S., Stasik S., Hanoun M., Schroeder T., Fransecky L., Steffen B. (2025). Validation of the revised 2022 European LeukemiaNet risk stratification in adult patients with acute myeloid leukemia. Blood Adv..

[36] Hoff F.W., Blum W.G., Huang Y., Welkie R.L., Swords R.T., Traer E., Stein E.M., Lin T.L., Archer K.J., Patel P.A. (2024). Beat-AML 2024 ELN-refined risk stratification for older adults with newly diagnosed AML given lower-intensity therapy. Blood Adv..

[37] Zhang X., Xiao N., Du S., Anipindi M., Lee C.H., Yoshino T., Anand A., Xiao L., Kumar A. (2026). Artificial intelligence-based prognostic models in acute myeloid leukemia: Systematic review and meta-analysis. Blood Neoplasia.

[38] Yadav S.K., Joshi U., Hussein G., Warsame M., Liu B., Shrestha A., Krastev P., Korsapati H.R., Singh A. (2025). Evolving Paradigms in Acute Myeloid Leukemia: Personalized Approaches to Therapy Across Age and Risk Groups. Cancers.

[39] Alnoor F., Mukherjee S., Menon M.P., Ng D., Li P., Ohgami R.S. (2026). Molecular Pathology, Artificial Intelligence, and New Technologies in Hematologic Diagnostics: Translational Opportunities and Practical Considerations. Diagnostics.

[40] Kim H., Hur M., d’Onofrio G., Zini G. (2025). Real-World Application of Digital Morphology Analyzers: Practical Issues and Challenges in Clinical Laboratories. Diagnostics.

[41] Austin E.E., Do V., Nullwala R., Fajardo Pulido D., Hibbert P.D., Braithwaite J., Arnolda G., Wiles L.K., Theodorou T., Tran Y. (2021). Systematic review of the factors and the key indicators that identify doctors at risk of complaints, malpractice claims or impaired performance. BMJ Open.

[42] Xie W., Jiang X., Huang J., Qin M., Bi Z. (2025). Research advances in the adjunctive diagnosis of acute myeloid leukemia. Front. Oncol..

[43] Hameed M., Raja M.A.Z., Zameer A., Dar H.S., Alluhaidan A.S., Aziz R. (2025). Acute myeloid leukemia classification using ReLViT and detection with YOLO enhanced by adversarial networks on bone marrow images. Sci. Rep..

[44] Sun S., Yin Z., Van Cleave J.G., Wang L., Fried B., Bilal K.H., Lucas F.S., Isgor I., Webb D.C., Singi S. (2025). DeepHeme, a high-performance, generalizable deep ensemble for bone marrow morphometry and hematologic diagnosis. Sci. Transl. Med..

[45] Kowald A., Fung C.H., Moon J., Shibeeb S. (2025). The Diagnostic Performance of the Cellavision DC-1 Digital Morphology Analyser on Leukaemia Samples. Diagnostics.

[46] Ghete T., Kock F., Pontones M., Pfrang D., Westphal M., Höfener H., Metzler M. (2024). Models for the marrow: A comprehensive review of AI-based cell classification methods and malignancy detection in bone marrow aspirate smears. Hemasphere.

[47] Al-Obeidat F., Hafez W., Rashid A., Jallo M.K., Gador M., Cherrez-Ojeda I., Simancas-Racines D. (2025). Artificial intelligence for the detection of acute myeloid leukemia from microscopic blood images; a systematic review and meta-analysis. Front. Big Data.

[48] Cheng H., Ding J., Wang J., Xiao Y., Jin X., Zhang Y., Yang Y., Xu H., Cao X., Guo F. (2025). Predicting *RUNX1*::*RUNX1T1* genetic abnormalities in acute myeloid leukemia from bone marrow smears: Can artificial intelligence do better?. iScience.

[49] Bermejo-Peláez D., Mendoza Martínez A., Gómez Álvarez M., Hidalgo Soto M., Diez N., Brau D., Oños A., Rodríguez S., Postigo M., Linares M. (2025). Predicting genetic alterations in Acute Myeloid Leukemia through AI-based analysis of bone marrow morphology. Blood.

[50] Lewis J.E., Cooper L.A.D., Jaye D.L., Pozdnyakova O. (2024). Automated Deep Learning-Based Diagnosis and Molecular Characterization of Acute Myeloid Leukemia Using Flow Cytometry. Mod. Pathol..

[51] Lucas F., Cherian S., Linden M.A., Tashakori M. (2026). Flow Cytometry in Acute Myeloid Leukemia (AML): A Critical Tool for Accurate Diagnosis, Classification, and Monitoring. Clin. Chem..

[52] Wang B., Li Z., Niu R., Yang Y., Zhu C., Liu H. (2025). Construction of a prognostic hierarchical model of intermediate-risk acute myeloid leukemia based on machine learning. Digit. Health.

[53] Steinicke T.L., Benfatto S., Capilla-Guerra M.R., Monteleone A.B., Young J.H., Shankar S., Michaels P.D., Tsai H.K., Good J.D., Kreso A. (2025). Rapid epigenomic classification of acute leukemia. Nat. Genet..

[54] Chen Y., He L., Ianevski A., Nader K., Ruokoranta T., Linnavirta N., Miettinen J.J., Vähä-Koskela M., Vänttinen I., Kuusanmäki H. (2025). A Machine Learning–Based Strategy Predicts Selective and Synergistic Drug Combinations for Relapsed Acute Myeloid Leukemia. Cancer Res..

[55] Hybel T.E., Jensen S.H., Rodrigues M.A., Hybel T.E., Pedersen M.N., Qvick S.H., Enemark M.H., Bill M., Rosenberg C.A., Ludvigsen M. (2024). Imaging Flow Cytometry and Convolutional Neural Network-Based Classification Enable Discrimination of Hematopoietic and Leukemic Stem Cells in Acute Myeloid Leukemia. Int. J. Mol. Sci..

[56] Yahya D., Stoyanova M., Hachmeriyan M., Levkova M. (2025). Genomic Evaluation of AML-Main Techniques and Novel Approaches. J. Clin. Med..

[57] Ishtyaq Mahmud M., Banerjee T. (2026). Artificial Intelligence in genomics: A comprehensive survey of methods, resources, challenges, and prospects. Brief. Bioinform..

[58] Soleimani Samarkhazan H. (2025). Integrating multi-omics approaches in acute myeloid leukemia (AML): Advancements and clinical implications. Clin. Exp. Med..

[59] Yue Z., Zhang Z., Thanh Q., Nguyen T., Sembay Z., Chen H., Welner R., Chen J. (2026). AI-driven gene-sets, networks, pathways, and interactions analyses of multi-omics data. Prog. Mol. Biol. Transl. Sci..

[60] Cheng W.Y., Li J.F., Zhu Y.M., Lin X.J., Wen L.J., Zhang F., Zhang Y.L., Zhao M., Fang H., Wang S.Y. (2022). Transcriptome-based molecular subtypes and differentiation hierarchies improve the classification framework of acute myeloid leukemia. Proc. Natl. Acad. Sci. USA.

[61] Meggendorfer M., Walter W., Haferlach C., Kern W., Haferlach T. (2019). Challenging Blast Counts by Machine Learning Techniques Genome Sequencing for Discriminating AML and MDS. Blood.

[62] Thakur R., Thieme S., Dempster M., Chaudhary N., Mishra S., Cronin T., Green S., Thompson J., Griffiths E., Sung P. (2025). Machine learning accurately predicts mortality in adult NPM1-mutant Acute Myeloid Leukemia using baseline clinical and genomic features. Blood.

[63] Sabile J.M.G., Zhang P., Parwani A.V., Chobrutsiky B., Gandhi A.P., Srisuwananukorn A. (2025). Toward Clinically Actionable Machine Learning and Artificial Intelligence Algorithms in Acute Leukemia: A Systematic Narrative Review. Acta Haematol..

[64] Dai Q., Yang Z., He B., Li Y. (2026). Isocitrate Dehydrogenase Inhibitors in Acute Myeloid Leukemia. Chem. Biodivers..

[65] Patel P., Pozdnyakova O. (2026). Advances in Digital Pathology and Artificial Intelligence in the Diagnosis of Myeloid Neoplasms. Hum. Pathol..

[66] Sharma J., Goel P. (2025). The Use of AI for Phenotype-Genotype Mapping. Methods Mol. Biol..

[67] Syrykh C., Bertoli S., Alliot J.M., Brousset P. (2026). Expectations and Limitations of Artificial Intelligence in Blood Cancer Diagnosis. Blood Cancer Discov..

[68] Zhang C., Lam B.D., Lucas F., Foy B.H. (2025). Machine Learning and Artificial Intelligence-Based Clinical Decision Support for Modern Hematology. Clin. Lab. Med..

[69] Siddiqui N.S., Klein A., Godara A., Buchsbaum R.J., Hughes M.C. (2022). Predicting In-Hospital Mortality After Acute Myeloid Leukemia Therapy: Through Supervised Machine Learning Algorithms. JCO Clin. Cancer Inform..

[70] Islam N., Reuben J.S., Dale J., Gutman J., McMahon C.M., Amaya M., Goodman B., Toninato J., Gasparetto M., Stevens B. (2022). Machine Learning-Based Exploratory Clinical Decision Support for Newly Diagnosed Patients with Acute Myeloid Leukemia Treated With 7 + 3 Type Chemotherapy or Venetoclax/Azacitidine. JCO Clin. Cancer Inform..

[71] DiNardo C.D., Jonas B.A., Pullarkat V., Thirman M.J., Garcia J.S., Wei A.H., Konopleva M., Döhner H., Letai A., Fenaux P. (2020). Azacitidine and Venetoclax in Previously Untreated Acute Myeloid Leukemia. N. Engl. J. Med..

[72] Pratz K.W., Jonas B.A., Pullarkat V., Thirman M.J., Garcia J.S., Döhner H., Récher C., Fiedler W., Yamamoto K., Wang J. (2024). Long-term follow-up of VIALE-A: Venetoclax and azacitidine in chemotherapy-ineligible untreated acute myeloid leukemia. Am. J. Hematol..

[73] Park S., Kim T.Y., Cho B.S., Kwag D., Lee J.M., Kim M., Kim Y., Koo J., Raman A., Kim T.K. (2024). Prognostic value of European LeukemiaNet 2022 criteria and genomic clusters using machine learning in older adults with acute myeloid leukemia. Haematologica.

[74] Islam N., Dale J.L., Reuben J.S., Sapiah K., Coates J.W., Markson F.R., Zhang J., Wu L., Gasparetto M., Stevens B.M. (2025). Development of a Dynamic Counterfactual Risk Stratification Strategy for Newly Diagnosed Patients with AML Treated with Venetoclax and Azacitidine. JCO Clin. Cancer Inform..

[75] Zhang C., Li J., Luo W., He S. (2025). AI-Assisted Detection for Early Screening of Acute Myeloid Leukemia Using Infrared Spectra and Clinical Biochemical Reports of Blood. Bioengineering.

[76] Radakovich N., Cortese M., Nazha A. (2020). Acute myeloid leukemia and artificial intelligence, algorithms and new scores. Best Pract. Res. Clin. Haematol..

[77] de Leeuw D.C., Ossenkoppele G.J., Janssen J.J.W.M. (2022). Older Patients with Acute Myeloid Leukemia Deserve Individualized Treatment. Curr. Oncol. Rep..

[78] Chen J., Gale R.P. (2026). Physician-complementing artificial intelligence in haematology: Ushering in a new era. Leukemia.

[79] Eckardt J.N., Middeke J.M. (2025). Promises and challenges of artificial intelligence in haematological diagnostics. Br. J. Haematol..

[80] Xu R., Tian H., Zhao S., Gu S. (2025). Construction and validation of a risk prediction model for complications in patients with acute leukemia based on machine learning. Sci. Rep..

[81] Turki A.T., Mussetti A., Scheidler K.M., Kehrmann J., Crivello P., Hosch R., Nensa F., Polizzi S. (2025). Artificial Intelligence in Hematopoietic Stem Cell Transplantation Care and Complication Management. Acta Haematol..

[82] Alipourfard I., Shafaghat Z., Saharkhiz S., Farani M.R., Bakhtiyari S. (2026). Emerging Applications of CAR-T Cell Therapy in Overcoming Resistance and Expanding Targets in Hematologic Malignancies: Insights from Recent Research. Curr. Treat. Options Oncol..

[83] Niscola P., Gianfelici V., Pasqualini G., Barroso E.E., Giovannini M., Catalano G., Mazzone C., Noguera N.I. (2026). New horizons for hope of cure in acute myeloid leukemia through immunotherapy: A narrative review. Expert. Rev. Anticancer. Ther..

[84] Palmieri R., Maurillo L., Del Principe M.I., Venditti A., Buccisano F. (2025). Fitness in acute myeloid leukemia, state of the art and future directions. Curr. Opin. Pharmacol..

[85] Aydin S., Passera R., Cerrano M., Giai V., D’Ardia S., Iovino G., Dellacasa C.M., Audisio E., Busca A. (2023). Combining the HCT-CI, G8, and AML-Score for Fitness Evaluation of Elderly Patients with Acute Myeloid Leukemia: A Single Center Analysis. Cancers.

[86] Sorror M.L. (2013). How I assess comorbidities before hematopoietic cell transplantation. Blood.

[87] Ferrara F., Barosi G., Venditti A., Angelucci E., Gobbi M., Pane F., Tosi P., Zinzani P., Tura S. (2013). Consensus-based definition of unfitness to intensive and non-intensive chemotherapy in acute myeloid leukemia: A project of SIE, SIES, and GITMO group on a new tool for therapy decision making. Leukemia.

[88] Bhatt V.R., Uy G.L., Klepin H.D. (2024). Determining treatment tolerance and fitness for intensive chemotherapy in older adults with AML: A call to action. Blood.

[89] Agaronnik N., Sounack T., Sanyal A., DuMontier C., Lindvall C., Abel G. (2025). Using large language models to identify frailty among older adults with blood cancer. Blood.

[90] Fernandes Prabhu D., Gurupur V., Stone A., Trader E. (2025). Integrating Artificial Intelligence, Electronic Health Records, and Wearables for Predictive, Patient-Centered Decision Support in Healthcare. Healthcare.

[91] Birla M., Rajan Roy P.G., Gupta I., Malik P.S. (2025). Integrating Artificial Intelligence-Driven Wearable Technology in Oncology Decision-Making: A Narrative Review. Oncology.

[92] Zheng X., Zeng Z., van Schooten K.S., Yang Y. (2025). Machine Learning Approach for Frailty Detection in Long-Term Care Using Accelerometer-Measured Gait and Daily Physical Activity: Model Development and Validation Study. JMIR Aging.

[93] Liu J., Pandya S., Coppi A., Young H.P., Krumholz H.M., Schulz W.L., Gong G. (2026). Assessment of the integrity of real-time electronic health record data used in clinical research. PLoS ONE.

[94] Efficace F., Patriarca A., Luppi M., Potenza L., Caocci G., Tafuri A., Fazio F., Cartoni C., Petrucci M.T., Carmosino I. (2022). Physicians’ Perceptions of Clinical Utility of a Digital Health Tool for Electronic Patient-Reported Outcome Monitoring in Real-Life Hematology Practice. Evidence From the GIMEMA-ALLIANCE Platform. Front. Oncol..

[95] El-Jawahri A., Luskin M.R., Greer J.A., Traeger L., Lavoie M., Vaughn D.M., Andrews S., Yang D., Boateng K.Y., Newcomb R.A. (2023). Psychological mobile app for patients with acute myeloid leukemia: A pilot randomized clinical trial. Cancer.

[96] Kwag D., Cho B.S., Park S., Kim H.J. (2025). Objectively measured physical function predicts mortality in older adults with acute myeloid leukemia treated by lower-intensity therapy: A prospective cohort study. J. Geriatr. Oncol..

[97] McCollom J.W., Tsang M. (2025). Objective Performance Status: Enhancing Cancer Patient Fitness Assessment with Wearable Technology. JCO Oncol. Pract..

[98] Jensen-Battaglia M., Sohn M.B., Consagra W., Wang Y., Zhang Z., LoCastro M., Davis J., Buettner K., Mortaz S., El-Jawahri A.R. (2024). Trajectories of physical well-being among adults with acute myeloid leukemia. Blood Adv..

[99] Meyers C.A., Albitar M., Estey E. (2005). Cognitive impairment, fatigue, and cytokine levels in patients with acute myelogenous leukemia or myelodysplastic syndrome. Cancer.

[100] Niscola P., Giovannini M., Efficace F. (2026). The importance of better understanding symptom trajectories in the evolving treatment landscape of acute myeloid leukemia. Support. Care Cancer.

[101] Oliva E.N., Almeida A. (2025). Determining treatment pathways for older patients with acute myeloid leukemia: Patient and clinician perspectives. Expert. Rev. Hematol..

[102] Karahanoglu F.I., Jensen C.E., Cai X., Santamaria M., Psaltos D., Messere A., Demanuele C., Stone J., Tarachandani A., Adamowicz L. (2025). Improved functional assessment in cancer patients using home-based digital technologies. Sci. Rep..

[103] Peipert J.D., Efficace F., Pierson R., Loefgren C., Cella D., He J. (2022). Patient-reported outcomes predict overall survival in older patients with acute myeloid leukemia. J. Geriatr. Oncol..

[104] Adjei Boakye E., Nair M., Al-Antary N., Wilson C., Kerr K., Zatirka T.M., Hirko K.A., Elsiss F., Chang S.S., Movsas B. (2025). Exploratory analysis of electronic patient-reported outcomes collection: Comparing online and in-clinic modalities in cancer care. Qual. Life Res..

[105] Tonino R.P.B., Schipperus M.R., Zwaginga J.J. (2025). Toward Personalized Transfusion Strategies: The Emerging Role of Wearable Biosensors in Chronic Anemia Management. Transfus. Med. Rev..

[106] Warnecke E., Salvador Comino M.R., Kocol D., Hosters B., Wiesweg M., Bauer S., Welt A., Heinzelmann A., Müller S., Schuler M. (2023). Electronic Patient-Reported Outcome Measures (ePROMs) Improve the Assessment of Underrated Physical and Psychological Symptom Burden among Oncological Inpatients. Cancers.

[107] Qin Y., Pu X., Hu D., Yang M. (2024). Machine learning-based biomarker screening for acute myeloid leukemia prognosis and therapy from diverse cell-death patterns. Sci. Rep..

[108] Wang Y., Liu R., Zhang Y., Luo X., Yu C., Fang S., Tan N., Tang J. (2025). A Network-Driven Framework for Precision Prediction of Drug Response in Acute Myeloid Leukemia. Adv. Sci..

[109] Tercan B. (2026). Robust predictors for drug response of patients with acute myeloid leukemia. PLoS ONE.

[110] Eckardt J.N., Hahn W., Röllig C., Stasik S., Platzbecker U., Müller-Tidow C., Serve H., Baldus C.D., Schliemann C., Schäfer-Eckart K. (2024). Mimicking clinical trials with synthetic acute myeloid leukemia patients using generative artificial intelligence. npj Digit. Med..

[111] Zhang Y., Martinez J., Tercan B., Kuusanmäki H., Emmert-Streib F., Chandraseelan J.G., Farea A., Yli-Harja O., Heckman C.A., Gibbs D.L. (2025). Digital twin models for predicting venetoclax and azacitidine-induced neutropenia in patients with acute myeloid leukemia. npj Digit. Med..

[112] Reimann A.M., Schalk E., Mougiakakos D., Fischer T., Sager S. (2026). Exploring innovative G-CSF schedules in AML cytarabine-based consolidation through a digital twin study of white blood cell recovery. PLoS ONE.

[113] Zhou Q., Chen Z.H., Peng S. (2025). Evaluating the application of dynamic prediction models in oncological prognostic studies with repeated measurement predictors. npj Precis. Oncol..

[114] Kirti A., Prachi D., Khan A. (2025). Artificial intelligence enhanced prognostication in rare Acute Myeloid Leukemia subtypes with BCR::ABL1 and inv(3)/t(3;): A systematic review. Blood.

[115] Hantel A., Clancy D.D., Kehl K.L., Marron J.M., Van Allen E.M., Abel G.A. (2022). A Process Framework for Ethically Deploying Artificial Intelligence in Oncology. J. Clin. Oncol..

[116] Pham T. (2025). Ethical and legal considerations in healthcare AI: Innovation and policy for safe and fair use. R. Soc. Open Sci..

[117] Froicu E.M., Creangă-Murariu I., Afrăsânie V.A., Gafton B., Alexa-Stratulat T., Miron L., Pușcașu D.M., Poroch V., Bacoanu G., Radu I. (2025). Artificial Intelligence and Decision-Making in Oncology: A Review of Ethical, Legal, and Informed Consent Challenges. Curr. Oncol. Rep..

[118] Zhou S., Liu X., Xu Z., Zhan Z., Song M., Wang J., Liu S., Xu H., Zhang R. (2025). Mitigating Ethical Issues for Large Language Models in Oncology: A Systematic Review. JCO Clin. Cancer Inform..

[119] Boudi A.L., Boudi M., Chan C., Boudi F.B. (2024). Ethical Challenges of Artificial Intelligence in Medicine. Cureus.

[120] Turchi T., Prencipe G., Malizia A., Filogna S., Latrofa F., Sgandurra G. (2024). Pathways to democratized healthcare: Envisioning human-centered AI-as-a-service for customized diagnosis and rehabilitation. Artif. Intell. Med..

[121] Sassi Z., Eickmann S., Roller R., Osmanodja B., Burchardt A., Tretter M., Samhammer D., Dabrock P., Möller S., Budde K. (2025). Human-centered AI in healthcare: Empowering patients and support persons in clinical decision-making. BMC Med. Inform. Decis. Mak..

[122] Garibyan L., Monnier J., Haykal D. (2026). Human-AI collaboration: Preserving the human touch in a digital age. Presse Med..

